# Potassium transporter OsHAK17 may contribute to saline-alkaline tolerant mechanisms in rice (*Oryza sativa*)

**DOI:** 10.1007/s10265-024-01529-0

**Published:** 2024-03-01

**Authors:** Mami Nampei, Hiromu Ogi, Tanee Sreewongchai, Sho Nishida, Akihiro Ueda

**Affiliations:** 1 Graduate School of Integrated Sciences for Life, Hiroshima University, 1-4-4 Kagamiyama, Higashi-Hiroshima City, Hiroshima 739-8528 Japan; 2 Department of Agronomy, Faculty of Agriculture, Kasetsart University, 50 Ngam Wong Wan Road, Lat Yao, Chatuchak, 10900 Bangkok Thailand; 3 Faculty of Agriculture, Saga University, 1Honjo-Machi, Saga City, Saga 840-8502 Japan; 4 United Graduate School of Agricultural Sciences, Kagoshima University, 1-21-24, Korimoto, Kagoshima City, Kagoshima 890-0065 Japan

**Keywords:** K^+^ acquisition mechanisms, K^+^ transporter, OsHAK17, Rice, Saline-alkaline stress

## Abstract

Rice production is seriously affected by saline-alkaline stress worldwide. To elucidate the saline-alkaline tolerance mechanisms in a novel tolerant rice variety, Shwe Nang Gyi (SNG), we investigated ion accumulation in SNG and Koshihikari (KSH), which is a saline-alkaline sensitive rice variety, and the candidates for saline-alkaline inducible genes in SNG using RNA-seq. SNG had superior ion accumulation capacity, such as K and Zn, compared to KSH. In contrast, SNG accumulated the same level of Na content in its leaf blades as KSH despite the higher dry weight of the SNG leaf blades. We further found that the expression of numerous genes, including several K^+^ transporter/high-affinity K^+^ transporter/K^+^ uptake protein/K^+^ transporter (HAK/KUP/KT) family members, were upregulated in SNG, and that OsHAK17 and OsHAK21 expression levels in the roots were significantly higher in SNG than in KSH. Moreover, yeast complementation analysis revealed that OsHAK17 was involved in K^+^ uptake under high-Na conditions. These results suggested that SNG has an effective K^+^ acquisition system supported by OsHAK17 functioning in saline-alkaline environments.

**Supplementary Information:**

The online version contains supplementary material available at 10.1007/s10265-024-01529-0.

## Introduction

The productivity of agricultural products such as rice is negatively affected by salt-affected soils. Annual income loss from salt-affected irrigated areas amounts to US$ 12 billion, based mainly on crop yield losses (Ghassemi et al. 1995; Qadir et al. 2014). In particular, saline-sodic soils containing alkaline salts such as NaHCO_3_ are the most severe environments in salt-affected soils. A study by Kono and Nagasawa (2019) showed that rice yield decreased by more than 30% in saline-sodic soils compared with non-salt-affected soils in northeastern Thailand, while it decreased by 15% in saline soils and 22% in sodic soils. Hence, generating a new saline-alkaline tolerant rice cultivar is urgently needed to achieve rice cultivation in salinized paddy fields with high pH.

Saline-alkaline stress is a combination of osmotic, ionic, and alkaline stresses; thus, its impact on plants is much more severe than that of saline stress (Rao et al. 2023). It results in various symptoms such as growth inhibition, reduction of photosynthetic efficiency, over-accumulation of Na, deficiency of essential elements such as K, Fe, Zn, and Mn, and ROS imbalance (Chuamnakthong et al. 2019; Fu et al. 2018; Li et al. 2015; Nampei et al. 2021; Rao et al. 2023; Ye et al. 2019). In contrast, plants develop saline-alkaline tolerance mechanisms to resist such disadvantageous situations. The saline-alkaline tolerant rice, Dongdao-4, has a great ability for diterpenoid and phenylpropanoid biosynthesis, at least at the transcriptome level, and is highly efficient at acquiring Fe (Li et al. 2016, 2020). The saline-alkaline tolerant monocot *Puccinellia tenuiflora* upregulated the expression of genes involved in iron acquisition under bicarbonate stress conditions (Kobayashi et al. 2015). A recent study reported that new candidates for saline-alkaline tolerance genes, which are related to protein binding and flavonoid biosynthesis, were found in maize (Li et al. 2022). Despite the best efforts of scientists, many aspects of saline-alkaline tolerant mechanisms are still unknown because of their complexity.

Under saline stress, excess Na^+^ uptake disrupts Na^+^/K^+^ homeostasis in plants (Assaha et al. 2017; Mekawy et al. 2015). Plants exclude Na^+^ from the cytosol by developing Na^+^-transport mechanisms to cope with this situation. SOS1, a Na^+^/H^+^ antiporter localized on the plasma membrane, plays an important role in eliminating intercellular Na to the extracellular space (Fukuda et al. 2004; Shi et al. 2002). Na^+^ flowing in the xylem from the roots to the shoots is excluded via Class I high-affinity potassium transporters (HKT1), including OsHKT1;4 and OsHKT1;5, which are located in the plasma membrane of xylem parenchyma cells (Cotsaftis et al. 2012; Horie et al. 2009; Kobayashi et al. 2017; Ren et al. 2005; Suzuki et al. 2016). It is assumed that NHXs located in the tonoplasts compartmentalized cytosolic Na^+^ into the vacuoles (Fukuda et al. 1999, 2011; Gaxiola et al. 1999; Sriskantharajah et al. 2020; Yokoi et al. 2002). Rice subjected to saline-alkaline stress accumulates higher levels of Na than that under saline stress; hence, these Na^+^ exclusion and compartmentalization mechanisms are critical for survival in saline-alkaline environments. Chuamnakthong et al. (2019) reported that FL478, a high Na-tolerant variety, shows saline-alkaline tolerance, which may be due to the low Na concentration supported by *OsSOS1* and *OsHKT1;5* activities in the roots.

Potassium acquisition and transport mediated by the K^+^ transporter/high-affinity K^+^ transporter/K^+^ uptake protein (HAK/KUP/KT) family in plants is also one of the key factors for survival under saline stress conditions (Wang et al. 2021). Rice contains 27 HAK/KUP/KT family members, which are classified into five clusters based on their amino acid sequences (Nieves-Cordones et al. 2016). Several HAK/KUP/KT family members participate in salt tolerance mechanisms. For instance, the expression of *OsHAK16*, which encodes the OsHAK16 protein located at the plasma membrane, was upregulated under low K or salt stress conditions, and the knockout or overexpression line of OsHAK16 diminished or improved K^+^ uptake and salt tolerance, respectively (Feng et al. 2019). Under saline-alkaline stress conditions, *OsHAK16* was also highly expressed in the roots of the salt-tolerant rice variety Pokkali (Nampei et al. 2021). Shen et al. (2015) reported that OsHAK21 is localized in the plasma membrane and expressed in the xylem parenchyma and individual endodermal cells involved in maintaining Na^+^/K^+^ homeostasis under salt stress by mediating K^+^ absorption.

Previously, we screened 96 rice varieties under saline-alkaline stress conditions (50 mM Na + pH 8.0–9.0) and identified a saline-alkaline tolerant variety, Shwe Nang Gyi (SNG), and a saline-alkaline sensitive variety, Koshihikari (KSH) (Fig. S1) (The corresponding manuscript will be submitted shortly). In this study, to understand molecular mechanisms of saline-alkaline tolerance in rice, the differences between these two varieties were compared in ion accumulation under saline or saline-alkaline stress, and a comprehensive transcriptome analysis using SNG was conducted. Furthermore, the putative potassium transporter OsHAK17 was identified through complementation analysis using yeast transformants. Our findings provide new insights into saline-alkaline tolerance mechanisms in rice and partially contribute to improving rice productivity in salinized paddy fields.

## Materials and methods

### Preparation of plant materials

Two rice (*Oryza sativa* L.) varieties, a saline-alkaline tolerant variety (Shwe Nang Gyi (SNG)) and a saline-alkaline sensitive variety (Koshihikari (KSH)), were used as materials. These varieties were selected based on previous screenings. Rice seeds were sterilized and soaked in water for two days. After germination, the rice was hydroponically cultivated in modified half-strength Kimura B solution including the following nutrients: 0.18 mM (NH_4_)_2_SO_4,_ 0.27 mM MgSO_4_ 7H_2_O, 0.09 mM KNO_3_, 0.09 mM KH_2_PO_4_, and 0.18 mM Ca(NO_3_)_2_ 4H_2_O, 19 µM FeSO_4_ 7H_2_O (used instead of Fe-EDTA), 48 µM H_3_BO_4_, 9 µM MnSO_4_ 5H_2_O, 0.3 µM CuSO_4_ 5H_2_O, 0.7 µM ZnSO_4_ 7H_2_O, and 0.09 µM Na_2_MoO_4_ 2H_2_O. Four-week-old rice plants were moved into control, saline (50 mM NaCl, pH 5.5) or saline-alkaline conditions (20 mM NaCl + 30 mM NaHCO_3_, pH 8.0–8.3). The pH of the hydroponic solution was adjusted to the indicated value daily using 2N HCl or 2N NaOH, and the hydroponic solution was replaced every five days.

### Sampling

For physiological analysis, SNG and KSH plants exposed to each stress were sampled 0, 24, 48, 72, 120, and 240 h after stress treatments. The rice plants were separated into the roots, leaf sheaths, and leaf blades and dried for three days at 70 ℃. Four replicates were prepared for the physiological analysis. Material from the SNG plants subjected to control or saline-alkaline conditions for 24 h were divided into roots, leaf sheaths, and leaf blades, immediately frozen using liquid nitrogen, and kept at − 80 ℃ until just before they were used to extract RNA for RNA-seq. Mixture of three plants were used as one replicate, and two replicates were prepared. For real-time PCR analysis, root samples of SNG and KSH treated with control, saline, or saline-alkaline conditions for 24 h were harvested using the same methods as for the RNA-seq samples.

### Element analysis

The elemental content of the roots, leaf sheaths, and leaf blades of SNG and KSH were analyzed using an inductively coupled plasma-optical emission spectrometer (SPECTROGREEN FMX46, HITACHI, Tokyo, Japan). The digestion methods followed Wheal et al. (2011): dried samples (100 mg) triturated using a crusher were digested in a mixture of 2 mL of concentrated HNO_3_ and 0.5 mL of H_2_O_2_ at 80 ℃ for 30 min and 125 ℃ for 2 h. The sample volume was adjusted to 25 mL using milli-Q water.

### RNA extraction

For RNA-seq analysis, total RNA was extracted from frozen samples (400 mg) of the roots, leaf sheaths, and leaf blades of SNG using Sepaspol-RNA I Super G (NACALAI TESQUE, INC., Kyoto, Japan) and the RBC Total RNA Extraction Kit Mini (Plant) (RBC Bioscience, Birmingham, UK). The first step of the method using Sepasol-RNA I was as follows: 4 mL of Sepasol-RNA I was added to the powdered, frozen samples and incubated for 5 min at room temperature. Then, 800 μL of chloroform was added to the mixture and the mixture was centrifuged at 15,000 × *g* for 15 min at 4 ℃. The supernatant was mixed with 2 mL of 100% isopropanol and incubated for 10 min. Again, the mixture was centrifuged at 15,000 × *g* for 5 min at 4 ℃. The supernatant was discarded and 4 mL of 75% EtOH was added to the sample. The sample was centrifuged at 15,000 × *g* for 5 min at 4 ℃, and the EtOH was completely removed by pipetting. The pellet was dissolved in 100 μL of RNase-free H_2_O. The second step of RNA extraction was performed using the RBC Total RNA Extraction Kit Mini (Plant) according to the kit manufacturer’s instructions. RNA concentration and quality were analyzed at every step using a NanoDrop One spectrophotometer. For real-time PCR analysis, RNA was extracted using an RNA extraction kit.

### RNA-seq analysis

RNA sequencing and cleaning of the reads were performed at the Beijing Genomics Institute (BGI). The reference genome sequence and annotation data were downloaded from the rice annotation project (RAP) (https://rapdb.dna.affrc.go.jp/download/irgsp1.html). The clean data were mapped to the rice reference genome sequence (Os-Nipponbare-Reference-IRGSP-1.0) using HISAT2 (Kim et al. 2015). The mapped reads were quantified for each gene using HTSEQ (Anders et al. 2015) and normalized using the R package edgeR (Robinson et al. 2009). Low-signal data (CPM < 0.4) was removed. Differentially expressed genes (DEGs) were defined as genes that had an absolute value of log_2_-fold change (log_2_FC) ≥ 2 and false discovery rate (FDR) ≤ 0.05. GO enrichment analysis was performed using AgriGo v2.0 (http://systemsbiology.cau.edu.cn/agriGOv2/) (Tian et al. 2017).

### Reverse Transcription Reaction, qRT-PCR Using SYBR Green

Reverse transcription of the extracted total RNA (1 μg) was performed using a 5 × Master Mix (TOYOBO, Osaka, Japan). The reaction mixture was prepared according to the manufacturer’s instructions. Quantitative real-time PCR was carried out on each cDNA sample using THUNDERBIRD SYBR Green Master Mix (TOYOBO, Osaka, Japan) and an ABI Step One Real-Time PCR System (Applied Biosystems, Foster City, CA, USA)(Mekawy et al. 2018). *OsUBC* was used as an endogenous control gene in the roots. The reaction mixture contained 7.5 µL SYBR Mix, 1.5 µL forward primer, 1.5 µL reverse primer, 0.3 µL 50 × ROX reference dye, 1 µL cDNA, and 3.2 µL milli-Q H_2_O. The following cycling parameters were used: initial incubation at 95 °C for 1 min, followed by 40 cycles of denaturation at 95 °C for 15 s and extension at 60 °C for 60 s, and melting curve analysis. The relative gene expression levels were calculated using the comparative 2^−ΔΔCT^ method. Data represent the average of three technical replicates.

### Generating yeast transformants and yeast complementation analysis

Linear pDR195 and *OsHAK17* sequences were amplified by PCR from the cDNA of SNG, plasmid pDR195, using the primers listed in Table S1. *OsHAK17* was inserted downstream of the PMA1 promoter in plasmid pDR195 using the In-Fusion HD Cloning Kit. pDR195 and pDR195 with *OsHAK17* were invited into AB11c strain (W303 *ena1Δ::HIS3::ena4Δ, nha1Δ::LEU2, nhx1Δ::TRP1*)(Kinclova-Zimmermannova et al. 2006) by LiAc/SS carrier DNA/PEG method (Gietz and Schiestl 2007). For the complementation assay, the AB11c strain with pDR195 or pDR195-*OsHAK17* was grown in 10 mL of SD liquid medium with adenine in 50 mL flasks overnight. The liquid medium was adjusted to OD_600_ = 1.0, and 3 μL of its diluted liquid by distilled water was spotted on solid SD medium with adenine, the indicated concentration of NaCl at the indicated medium pH. The medium pH and K concentration of the SD medium was adjusted by 1 N KOH or 1 N KCl, respectively. For measurement of Na^+^ and K^+^ concentration in yeast, yeast transformants were pre-cultured overnight. Then, the culture solution was added to 10 mL of SD medium to bring OD_600_ = 0.005. The yeast transformants in SD medium were cultured until OD_600_ = 0.6–0.8, and 0 or 200 mM NaCl and 10 mM KCl were added to the cell culture to measure Na^+^ and K^+^ concentrations in yeast. Yeast cells were collected 48 h after stress treatment, quickly washed with distilled water three times, and then dried for one day. Na and K in the dried yeast transformants were extracted using boiling water, and their concentrations were measured using a flame photometer (ANA-135, TOKYO PHOTOELECTRIC CO.,LTD., Tokyo, Japan).

### Statistical analysis

Two-way analysis of variance (ANOVA) and the Tukey–Kramer test were performed using R software to detect statistical differences. To compare the statistical differences between the two rice varieties, F-tests and Student’s *t* test were used in Microsoft Excel.

## Results

### Plant growth

**Fig. 1:**
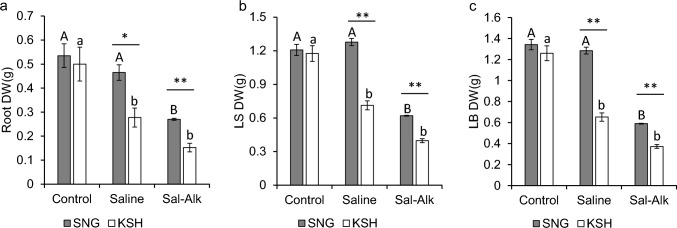
The dry weight of the roots (**a**), leaf sheaths (**b**), and leaf blades (**c**) of saline-alkaline tolerant variety (SNG) and saline-alkaline sensitive variety (KSH) under control (0 mM Na + pH 5.5), saline (50 mM Na + pH 5.5) and saline-alkaline (Sal-Alk) (50 mM Na + pH 8.0–8.3) conditions at 240 h. The data is represented as the means of four replicates ± SE. Dry weight was compared between the two rice varieties or three different treatments (*p < 0.05, **p < 0.01)

The saline-alkaline tolerant variety, SNG, and the sensitive variety, KSH, were selected in our previous screening. The dry weight of rice subjected to control, saline, and saline-alkaline treatments for 240 h was measured to identify the effect of saline-alkaline stress on the two rice genotypes. In both varieties, the dry weights of the roots, leaf sheaths, and leaf blades under saline-alkaline conditions dramatically decreased in the three treatments (Fig. 1). In SNG, the dry weights of the roots, leaf sheaths, and leaf blades decreased by 49.6%, 48.7%, and 56.1%, respectively, compared to those of the control. In KSH, the dry weights of the roots, leaf sheaths, and leaf blades decreased by 69.5%, 66.2%, and 70.4%, respectively, compared to those of the control. The dry weight of SNG under saline conditions was not significantly reduced compared to that of the control, whereas that of KSH was remarkably lower than that of the control. The dry weights of the roots, leaf sheaths, and leaf blades of SNG were 1.7, 1.8, and 2.0-fold higher than those of KSH under saline conditions, respectively.

### Element analysis

**Fig. 2:**
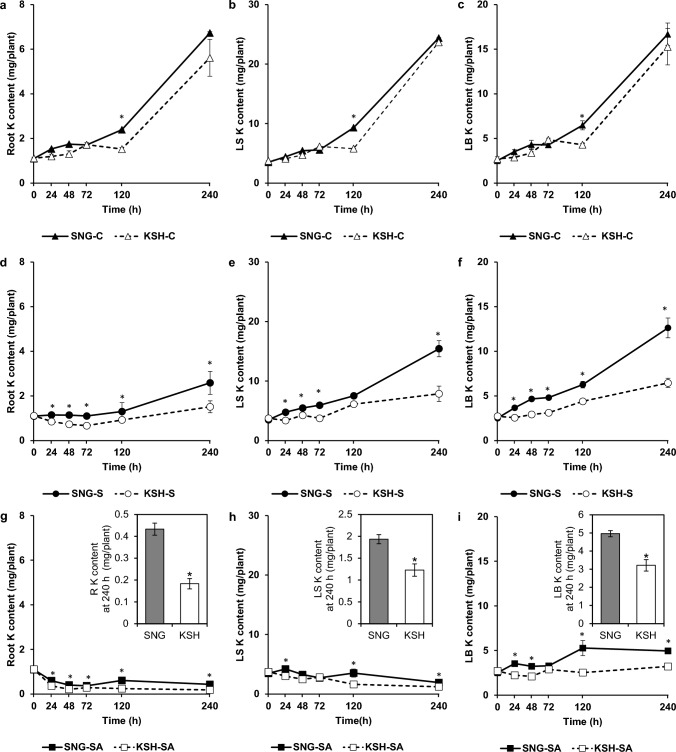
Potassium contents under control (0 mM Na + pH 5.5) (**a**–**c**), saline (50 mM Na + pH 5.5) (**d**–**f**), and saline-alkaline (50 mM Na + pH 8.0–8.3) (**g**–**i**) conditions in the roots (**a**, **d**, **g**), leaf sheaths (**b**, **e**, **h**), and leaf blades (**c**, **f**, **i**) of saline-alkaline tolerant variety (SNG) and saline-alkaline sensitive variety (KSH). The data is represented as the means of four replicates ± SE. The potassium contents were compared between the two rice varieties at each sampling time (*p* < 0.05)

Previous studies have reported that Na and K accumulations under saline-alkaline conditions were remarkably increased and decreased, respectively, compared with those under saline conditions (Chuamnakthong et al. 2019; Nampei et al. 2021). We examined temporal changes in Na and K contents in the roots, leaf sheaths, and leaf blades and the contents of other essential elements (Ca, Mg, P, Mn, Fe, Cu, and Zn) at the endpoint of the stress treatments in both rice varieties exposed to the control, saline, and saline-alkaline treatments to determine differences in the effects of saline-alkaline stress on nutrient accumulation between SNG and KSH. K content under the control conditions in SNG and KSH increased with growth (Fig. 2a–c). The elevation of K content under saline conditions in both rice varieties was gradual compared with that under control conditions (Fig. 2d–f). Comparing SNG and KSH under saline conditions, K contents in the roots, leaf sheaths, and leaf blades of SNG were 1.7-, 2.0-, and 2.0-fold higher than those of KSH, respectively, 240 h after the stress treatments. The K content in the roots and leaf sheaths under saline-alkaline conditions was reduced by 60% and 45% in SNG and 84% and 67% in KSH, respectively, compared to those at 0 h of stress initiation (Fig. 2g–i). Leaf blade K content in SNG under saline-alkaline conditions increased twofold, while those in KSH increased only 1.2-fold from the beginning of the stress treatment. Moreover, K in the roots, leaf sheaths, and leaf blades of SNG was 2.4-, 1.6-, and 1.5-fold higher than that in KSH 240 h after the saline-alkaline stress treatment.

**Fig. 3:**
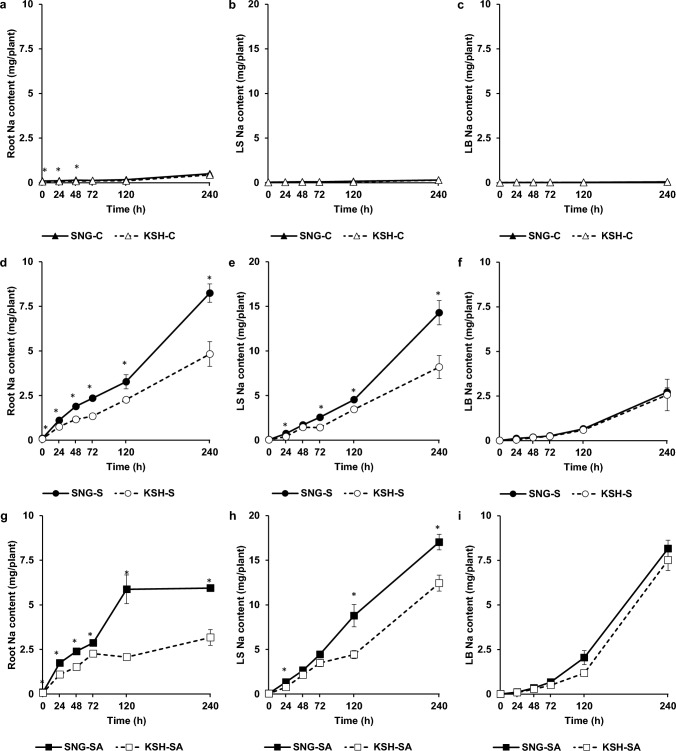
Sodium contents under control (0 mM Na + pH 5.5) (**a**–**c**), saline (50 mM Na + pH 5.5) (**d**–**f**), and saline-alkaline (50 mM Na + pH 8.0–8.3) (**g**–**i**) conditions in the roots (**a**, **d**, **g**), leaf sheaths (**b**, **e**, **h**) and leaf blades (**c**, **f**, **i**) of saline-alkaline tolerant variety (SNG) and saline-alkaline sensitive variety (KSH). The data is represented as the means of four replicates ± SE. The sodium contents were compared between the two rice varieties at each sampling time (*p* < 0.05)

 Na content in all parts of SNG and KSH under control conditions was significantly lower than that under saline or saline-alkaline conditions, and there was little difference between the two varieties (Fig. 3a–c). Under saline conditions, a sharp elevation of root and leaf sheath Na content in SNG from 0 h of stress treatment to 240 h was observed compared to that of KSH, and the Na content in the roots and leaf sheaths in SNG at the endpoint of the stress was 1.7-fold higher than that of KSH (Fig. 3d, e). In contrast, LB Na content did not differ between tolerant and sensitive rice varieties (Fig. 3f). Similar trends in the Na content under saline conditions were observed under saline-alkaline conditions. Na content in the roots and leaf sheaths in SNG rapidly increased from the beginning of stress to the endpoint compared with those in KSH (Fig. 3g, h), while in leaf blades, both rice varieties of Na contents were dramatically increased, and there was no significant difference between the two varieties (Fig. 3i). In addition, under saline-alkaline at 240 h after stress treatments, the Na/K ratio in the roots, leaf sheaths and leaf blades of SNG saline-alkaline conditions tended to be lower than those of KSH (Fig. S2).

**Fig. 4:**
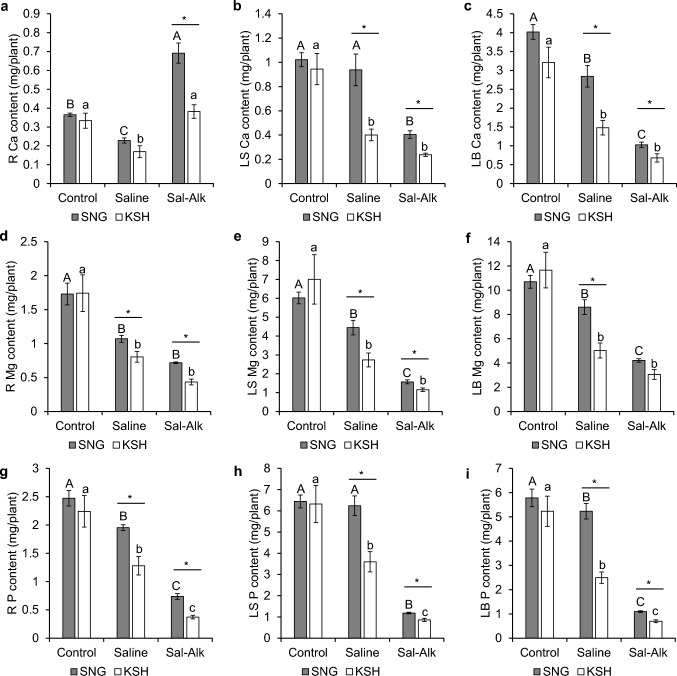
Calcium (**a**, **b**, **c**), Magnesium (**d**, **e**, **f**) and phosphorus (**g**, **h**, **i**) contents in the roots (**a**, **d**, **g**), leaf sheath (**b**, **e**, **h**), and leaf blade (**c**, **f**, **i**) of saline-alkaline tolerant variety (SNG) and saline-alkaline sensitive variety (KSH) under control (0 mM Na + pH 5.5), saline (50 mM Na + pH 5.5), and saline-alkaline (50 mM Na + pH 8.0–8.3) conditions. The data is represented as the means of four replicates ± SE. The ion contents were compared between the two rice varieties or three different treatments (*p* < 0.05)

The Ca content under saline conditions was lower than that under control conditions in the roots of both varieties (Fig. 4a). Meanwhile, only the Ca content in the roots of SNG increased under saline-alkaline conditions and was 1.8-fold higher than that of KSH. The Ca content in leaf sheaths and leaf blades tended to notably decrease in the order of saline-alkaline and saline conditions (Fig. 4b, c). The Mg and P contents in the roots, leaf sheaths, and leaf blades were also reduced in the order of saline-alkaline and saline conditions, and those of SNG were significantly higher than those of KSH under saline and saline-alkaline conditions (Fig. 4d–i).

**Fig. 5:**
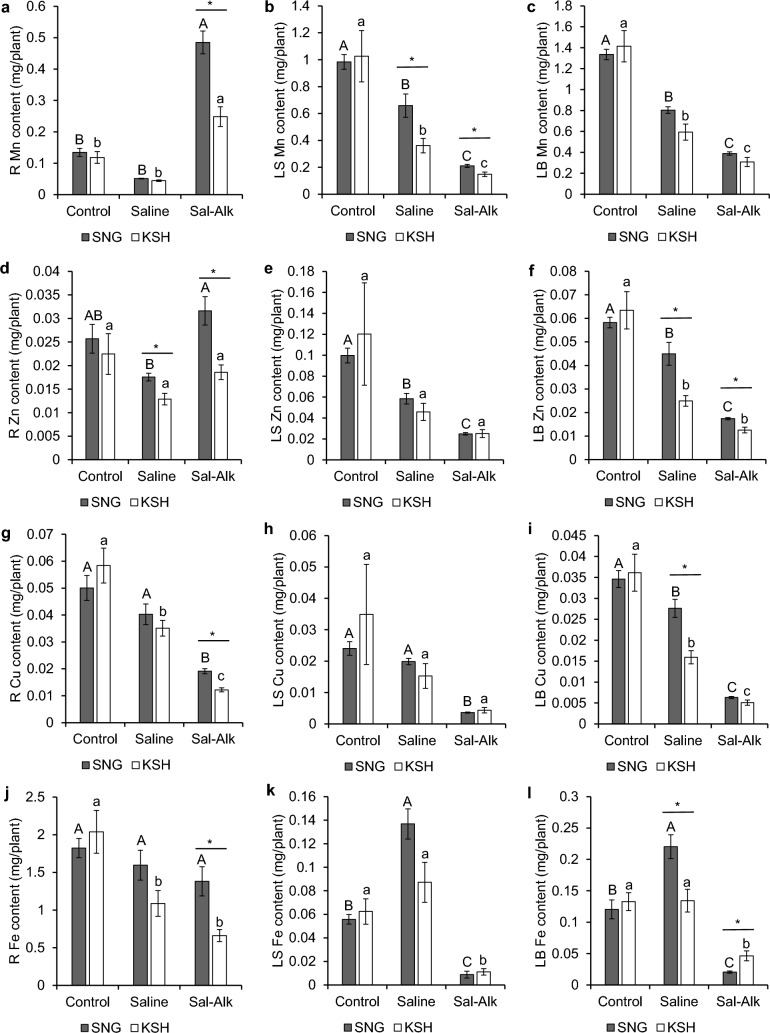
Manganese (**a**, **b**, **c**), zinc (**d**, **e**, **f**), copper (**g**, **h**, **i**), and iron (**j**, **k**, **l**) contents in the roots (**a**, **d**, **g**, **j**), leaf sheath (**b**, **e**, **h**, **k**), and leaf blade (**c**, **f**, **i**, **l**) of saline-alkaline tolerant variety (SNG) and saline-alkaline sensitive variety (KSH) under control (0 mM Na + pH 5.5), saline (50 mM Na + pH 5.5), and saline-alkaline (50 mM Na + pH 8.0–8.3) conditions. The data is represented as the means of four replicates ± SE. The ion contents were compared between the two rice varieties or three different treatments (*p* < 0.05)

Mn and Zn contents (0.48 mg and 0.031 mg, respectively) in the roots of SNG under saline-alkaline conditions were significantly higher than those under the control and saline conditions (Fig. 5a, d). The Mn content in the roots of KSH under saline-alkaline conditions was the highest under all conditions (Fig. 5a), and the Zn content of KSH under saline-alkaline conditions was similar to that under control conditions (Fig. 5d). In both varieties, Cu and Fe contents in the roots and Mn, Zn, Cu, and Fe contents in the leaf sheaths and leaf blades were the lowest under saline-alkaline conditions (Fig. 5b, c, e, f, g–l). The Fe content in the leaf sheaths and blades under saline conditions tended to be higher than that under control conditions, especially in SNG (Fig. 5 k, l). Under saline-alkaline conditions, the contents of the four divalent metals in the roots were significantly higher in SNG than in KSH (Fig. 5a, d, g, j), while the Mn content in the leaf sheaths and Zn content in the leaf blades in SNG were 1.4-fold higher than those in KSH (Fig. 5b, f). In contrast, the Fe content in the leaf blades of KSH was 2.2-fold higher than that in SNG under saline-alkaline conditions (Fig. 5l).

### Transcriptome analysis by RNA-seq and Gene Ontology enrichment analysis

**Fig. 6:**
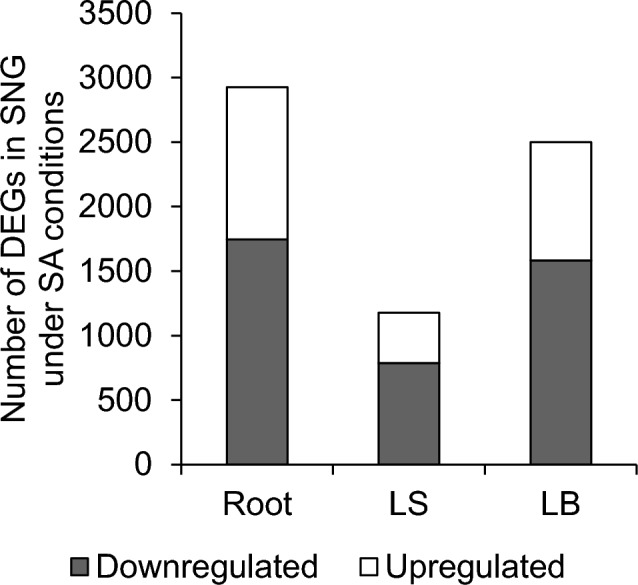
Results of differentially expressed genes in the roots, leaf sheath, and leaf blade of SNG under control and saline-alkaline (50 mM Na + pH 8.0–8.3) conditions as identified in RNA-seq. The differentially expressed genes were obtained by comparing comprehensive gene expressions of SNG under control and saline-alkaline conditions

RNA expression was comprehensively analyzed using RNA-seq to understand the initial response of the saline-alkaline tolerant rice variety SNG to saline-alkaline stress. In the roots, leaf sheath and leaf blades of SNG exposed to saline-alkaline stress for 24 h, 2926, 1178, and 2499 genes were differentially expressed, including 1745, 787, and 1583 downregulated and 1181, 391, and 916 upregulated DEGs, respectively (Fig. 6).

Gene Ontology (GO) enrichment analysis was performed to obtain an overview of the response of SNG to saline-alkaline stress. In total, 22 GO terms were enriched for the downregulated DEGs in the roots (Fig. S3). The top 3 enriched GO terms of downregulated DEGs in the roots were “oxidation–reduction,” “photosynthesis,” and “response to oxidative stress” in order (Fig. S3a). Besides, enriched GO terms in downregulated DEGs in the roots included “lipid transport,” “lipid metabolic process,” “carbohydrate metabolic process and cellular carbohydrate metabolic process,” and “lignin catabolic process.” In total, 51 GO terms were enriched for upregulated DEGs in roots, including “diterpene phytoalexin metabolic process,” “phytoalexin metabolic process,” “protein amino acid phosphorylation,” “defense response,” “isoprenoid metabolic process,” “chitin catabolic process,” and “tryptophan metabolic process” (Fig. S3b). “Transition metal ion transport,” “metal ion transport,” and “zinc ion transport” GO terms were also enriched in the upregulated DEGs. For the down- and upregulated DEGs, 25 and 17 GO terms in the leaf sheath and 50 and 5 GO terms in leaf blade were significantly enriched, respectively (Fig. S4). Similar to GO terms of upregulated DEGs, several GO terms such as “chitin catabolic process” and “defense response” were enriched in upregulated DEGs in leaf sheaths, whereas enriched GO terms such as “diterpenoid metabolic process” and “isoprenoid metabolic process” enriched in upregulated DEGs in the roots were included in GO terms of downregulated DEGs in the leaf sheaths and leaf blades (Figs. S3b, S4a-c). For upregulated DEGs in leaf blades, “protein complex assembly,” “regulation of protein complex assembly,” protein complex biogenesis,” “regulation of cellular component biogenesis,” and “cellular protein complex assembly” were observed as the enriched GO terms (Fig. S4d).

Furthermore, to observe ion transport activities in SNG under saline-alkaline conditions, we focused on the expression of Na^+^, K^+^, and divalent metal transporters. Under saline-alkaline stress, the expression of *OsNHX1* and *OsHKT1;4* in the roots and *OsHKT1;3* in the leaf blades were significantly downregulated, and no other Na^+^ transporter genes were differentially expressed in the RNA-seq analysis (Table S2). This indicated that Na^+^ transporter mechanisms were not activated in SNG under the initial stage of saline-alkaline stress. Meanwhile, five (*OsHAK11*, *OsHAK13*, *OsHAK17*, *OsHAK21*, and *OsHAK24*) out of 27 rice *HAK/KUP/KT* family members were highly upregulated, and two genes (*OsHAK7* and *OsHAK22*) were downregulated in the roots of SNG (Table 1). In particular, the log_2_ fold changes in *OsHAK17* and *OsHAK21* expressions were 3.97 and 6.72, respectively, indicating that these genes might be more highly induced by saline-alkaline stress than others. The expression of *OsHAK7* in the leaf sheath and *OsHAK5* and *OsHAK8* in the leaf blades were identified as downregulated DEGs, and *OsHAK12* in the leaf blade was identified as an upregulated DEG in the leaf blade under saline-alkaline conditions. In addition, the expression of *OsHKT2;4*, which encodes OsHKT2;4 and mainly transports K^+^ under high-sodium conditions, was significantly upregulated in leaf sheaths and blades (Table 2).

**Table 1:** Relative expression level of K^+^ transporter genes in the roots, leaf sheaths, and leaf blades of saline-alkaline tolerant variety SNG under saline-alkaline conditions

Gene	Roots		Leaf sheaths		Leaf blades	
	log_2_FC	FDR	log_2_FC	FDR	log_2_FC	FDR
*OsHAK1*	− 0.85	0.00	0.02	1.00	− 0.50	0.16
*OsHAK2*	− 0.35	0.36	0.21	0.91	0.30	0.52
*OsHAK3*	− 0.47	1.00	0.80	1.00	− 2.87	0.55
*OsHAK4*	− 0.82	0.91	-2.50	0.29	0.62	1.00
*OsHAK5*	0.88	0.24	-1.02	0.41	− 1.51	0.02
*OsHAK6*	5.87	0.15	1.19	1.00	0.00	1.00
*OsHAK7*	− 1.33	0.00	-1.97	0.00	− 0.99	0.00
*OsHAK8*	− 0.01	1.00	0.08	1.00	− 1.29	0.00
*OsHAK9*	− 0.47	0.07	-0.02	1.00	− 0.32	0.43
*OsHAK10*	0.07	1.00	0.52	0.07	0.69	0.00
*OsHAK11*	1.42	0.00	0.35	0.38	− 0.29	0.42
*OsHAK12*	− 0.53	0.05	0.40	0.34	1.43	0.00
*OsHAK13*	1.21	0.00	− 0.10	1.00	− 0.82	0.05
*OsHAK14*	0.09	1.00	0.49	0.45	0.65	0.13
*OsHAK15*	− 0.31	0.23	-0.07	1.00	− 0.50	0.03
*OsHAK16*	0.41	0.33	0.33	0.84	− 0.40	0.36
*OsHAK17*	3.97	0.00	0.57	0.24	0.23	0.80
*OsHAK18*	− 0.07	1.00	0.34	0.32	− 0.29	0.31
*OsHAK19*	0.33	1.00	− 1.09	0.17	0.04	1.00
*OsHAK20*	2.76	1.00	0.00	1.00	3.44	1.00
*OsHAK21*	6.72	0.00	2.56	1.00	0.00	1.00
*OsHAK22*	− 1.70	0.00	− 0.28	0.93	-0.32	0.66
*OsHAK23*	− 0.44	0.15	0.68	0.04	0.35	0.38
*OsHAK24*	1.08	0.01	0.29	1.00	0.33	0.85
*OsHAK25*	0.67	0.00	− 0.01	1.00	0.47	0.06
*OsHAK26*	5.15	0.25	− 0.65	1.00	− 2.90	0.45
*OsHAK27*	0.64	0.62	− 0.30	0.67	− 0.38	0.24

**Table 2:** Relative expression level of K^+^/Na^+^ co-transporter genes in the roots, leaf sheaths, and leaf blades of saline-alkaline tolerant variety SNG under saline-alkaline conditions

Gene	Roots		Leaf sheaths		Leaf blades	
	log_2_FC	FDR	log_2_FC	FDR	log_2_FC	FDR
*OsHKT2;1*	− 1.13	0.01	0.79	0.82	1.53	0.36
*OsHKT2;3*	− 5.34	0.23	0.77	1.00	− 0.24	1.00
*OsHKT2;4*	2.76	1.00	2.25	0.03	1.05	0.04

In contrast, nine divalent metal transporter genes (*OsNramp5, OsNramp6, OsZIP4, OsZIP5, OsZIP8, OsZIP9 OsZIP10, OsIRT1*, *and OsIRT2*) were significantly expressed in the roots of SNG under saline-alkaline conditions, whereas *OsZIP1* in the leaf sheath and leaf blades and *OsNramp5* and *OsIRT2* in leaf blades were observed as downregulated DEGs (Table S3).

### Gene expression of K^+^ transporters

**Fig. 7:**
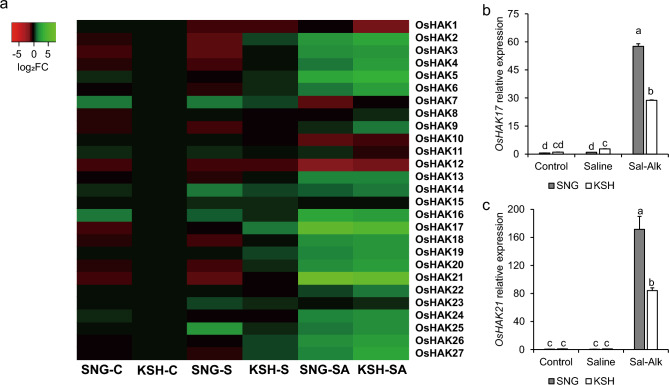
(**a**) Heatmap of real-time PCR analysis of expression of rice *KUP/HAK/KU* family genes in the roots of the tolerant (SNG) and sensitive (KSH) rice varieties under control (0 mM Na + pH 5.5), saline (50 mM Na + pH 5.5), and saline-alkaline (50 mM Na + pH 8.0–8.3) conditions. The details of the expression levels of *OsHAK17* (**b**) and *OsHAK21* (**c**) in SNG and KSH under control, saline, and saline-alkaline stress conditions. The data is represented as the means of three replicates ± SE. Significant differences are shown as different letters (*p* < 0.05)

The results of element contents and comprehensive gene expression analysis using RNA-seq showed that K contents in SNG were significantly higher than those of KSH, and some of the *HAK/KUP/KT* family genes were identified as upregulated DEGs in SNG. Therefore, we compared the differences in the expression of *HAK/KUP/KT* family genes between SNG and KSH under control, saline, and saline-alkaline stress conditions. The results of real-time PCR analysis showed that 15 members of the *HAK/KUP/KT* genes, including *OsHAK13, OsHAK17, OsHAK21*, and *OsHAK24*, which were upregulated DEGs (Table 1), were significantly expressed under saline-alkaline stress conditions in both rice varieties (Fig. 7a). In particular, the expression of *OsHAK17* and *OsHAK21* under saline-alkaline stress conditions increased by 58-fold and 172-fold in SNG and by 29-fold and 84-fold in KSH, respectively, compared to that of KSH under control conditions (Fig. 7b, c). This suggested that OsHAK17 and OsHAK21 are saline-alkaline inducible genes and are involved in the K^+^ acquisition system in SNG under saline-alkaline stress conditions.

### Function of OsHAK17 in yeast

A past study showed OsHAK21 functions and its contribution to salt tolerant mechanisms in rice (Shen et al. 2015). As for OsHAK17, Wang et al. (2020) revealed that *oshak4/17* double mutant rice showed high Na sensitivity, however, the function of OsHAK17 alone has not still been identified yet. Therefore, we focused on OsHAK17 and investigated the OsHAK17 functions under saline-alkaline conditions by performing yeast complementation assay.

**Fig. 8:**
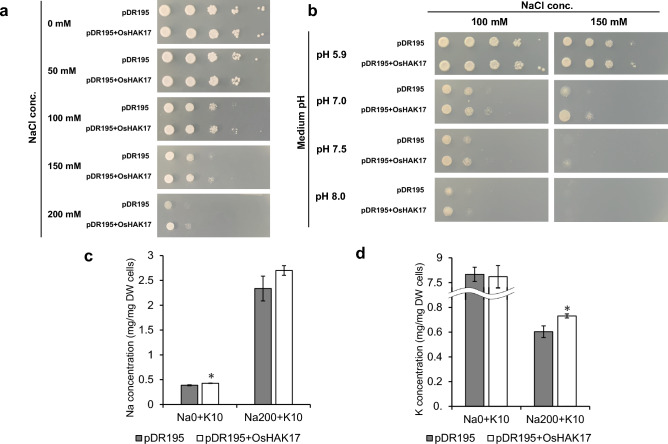
(**a**, **b**) Yeast complementation assay using AB11c strain with pDR195 or pDR195 + *OsHAK17.* Each yeast transformant was grown under SD solid medium with indicated NaCl concentrations for 2 days (**a**), or 100 or 150 mM NaCl concentration and indicated medium pH for 3 days (**b**). **c**, **d** Na (**c**) and K (**d**) concentrations of AB11c strain harboring pDR195 empty vector or OsHAK17. Yeast transformants were cultured in SD medium with 0 mM or 200 mM NaCl and 5 mM KCl. Four independent colonies were used as replicates. The data is represented as the means of three replicates ± SE. The ion contents were compared between the two rice varieties or three different treatments (*p* < 0.05)

Low K sensitive yeast strain, CY162, harboring the full-length *OsHAK17* cDNA was cultured in SD medium with various Na concentration (50, 100, 150 mM). However, no significant difference was observed in yeast CY162 transformants expressing *OsHAK17*. Ectopic expression of *OsHAK17* under low K conditions (in arginine-phosphate medium) did not also recover low K sensitive phenotype in CY162 (data not shown). The AB11c strain, with or without the full-length *OsHAK17* sequence inserted into pDR195, was grown in SD solid medium with the indicated NaCl concentrations. Yeasts expressing OsHAK17 grew more than those expressing the empty vector under 100, 150, and 200 mM NaCl conditions (Fig. 8a). Under 100 mM Na + pH 7.0, 7.5 or 8.0 conditions, and 150 mM Na + pH 7.0 or 7,5 conditions, yeast harboring OsHAK17 had greater growth compared to control (Fig. 8b). Contrarily, yeast did not grow under 150 mM Na + pH 8.0 conditions. To further clarify the Na^+^ or K^+^ transport function of OsHAK17, the Na and K concentrations in yeast cells grown in SD medium with the indicated Na and/or K concentrations were measured. Under 0 mM Na + 10 mM K conditions, the yeast cells harboring OsHAK17 contained slightly higher Na concentration (0.43 mg/mg DW cells) than the yeast with empty vector (0.39 mg/mg DW cells), while no significant differences in K concentrations were found between two yeast transformants (Fig. 8c, d). In contrast, under SD medium with 200 mM Na + 10 mM K conditions, Na concentrations in the transformants with empty vector and OsHAK17 were similar, whereas K concentrations in transformants harboring OsHAK17 were significantly higher (0.73 mg/mg DW cells) than those in empty vector control (0.60 mg/mg DW cells) (Fig. 8c, d). These results were consistent with those of the yeast growth assay, suggesting that OsHAK17 may contribute to K^+^ uptake under high-Na conditions but not under low-Na conditions.

## Discussion

Rice production is adversely affected by saline-alkaline stress, which threatens a stable food supply. Understanding saline-alkaline tolerance mechanisms is necessary for generating new rice cultivars that can be cultivated in salt-affected soils with high pH. In this study, we proposed new saline-alkaline tolerance mechanisms in a novel saline-alkaline tolerant rice variety by ionome and comprehensive and quantitative gene expression analyses.

K is the most abundant inorganic cation in plants such as rice, and K deficiency strongly regulates plant growth. Under saline-alkaline stress, which causes serious K deficiency in plants, Na^+^ exclusion from shoots involving OsSOS1 and class I OsHKTs, Na^+^ compartmentalization to the vacuole involving OsNHXs, and activation of K^+^ acquisition systems involving OsHAKs are cardinal survival strategies (Rao et al. 2023). In the current study, K was more highly accumulated in SNG rice than in KSH under saline-alkaline conditions. Notably, the K content in the LB of SNG was higher than that of KSH with the passage of the saline-alkaline stress treatment (Fig. 2). In contrast, the Na content of SNG was significantly higher in the roots and leaf sheaths than in KSH, whereas in the leaf blades, the dry weight of SNG was much higher than that of KSH; nevertheless, the Na content was the same as that of KSH (Figs. 1c, 3). These findings suggest that SNG rice has a great ability for K^+^ acquisition from the rhizosphere, K^+^ translocation to shoots, and Na^+^ exclusion from shoots. The results of comprehensive and quantitative gene expression analyses showed that SNG subjected to saline-alkaline stress enhanced K^+^ transport gene expressions, such as *OsHAK21* and *OsHAK17*, in the roots (Table 1, Fig. 7). However, no genes related to Na^+^ transport were induced throughout the plant. Based on these findings, SNG is thought to mainly activate the K^+^ transport system during the initial stage of stress.

The HAK/KUP/KT family members were classified into five clades, and OsHAK17 and OsHAK21 were categorized into Clade IV and Clade I, respectively (Li et al. 2018b; Nieves-Cordones et al. 2016). Compared to the HAK/KUP/KT family genes in Clade I, whose functions have been relatively well characterized, only a few genes in Clade IV were analyzed (Benito et al. 2012; Takahashi et al. 2007; Wang et al. 2020). OsHAK21 is a potassium transporter localized to the plasma membrane and is expressed in the xylem parenchyma cells and individual endodermal cells in rice (Shen et al. 2015). *OsHAK21* expression was dramatically induced by high NaCl, and OsHAK21 played an important role in K^+^ uptake in the roots and the maintenance of Na/K homeostasis in whole rice plants under salt stress conditions (Okada et al. 2018; Shen et al. 2015). Additionally, the past study reported that *OsHAK21* was also upregulated under saline-alkaline and alkaline with pH > 11.0 conditions (Li et al. 2018a; Lin et al. 2022). Contrarily, in the present study, the yeast complementation assay showed that the growth of the transformants harboring *OsHAK17* was better than that of control under severe saline stress and saline-alkaline conditions and K concentrations in yeast with OsHAK17 were higher than those in the empty vector controls under saline stress conditions (Fig. 8). Overall, K accumulation in yeast mutants was facilitated by OsHAK17 under high Na and high K conditions, indicating that OsHAK17 may function as a low affinity K^+^ transporter driven by Na^+^.

In both of SNG and KSH plants, OsHAK17 probably play important roles in K^+^ acquisition system since they were significantly expressed under saline-alkaline stress, but their expressions were quite higher in SNG than KSH (Fig. 7b). Based on the facts that K content in SNG was higher than that in KSH under saline-alkaline conditions (Fig. 2g–i), and OsHAK17 functioned as K^+^ transporter under saline-alkaline stress conditions (Fig. 8b), K^+^ acquisition system supported by OsHAK17 may actively work more in SNG than KSH under saline-alkaline stress.

In the current study, OsHAK17 were highly expressed under saline-alkaline stress but not under saline conditions. However, considering the facts that yeast transformants harboring *OsHAK17* grew better than control yeast under high Na conditions (Fig. 8a, b), it is estimated that OsHAK17 probably also contribute to salt tolerant mechanisms in SNG and KSH. However, mild Na^+^ influx into rice under saline conditions than saline-alkaline conditions (Fig. 3d–i) did not trigger increased expression (especially the timing of upregulation of the genes) of *OsHAK17* under saline condition. Further analysis is required to establish the mechanisms of saline-alkaline tolerance mediated by OsHAK17 function by conducting molecular biological approaches such as overexpression and knock down of the *OsHAK17* gene together with analyses of subcellular and spatial localization at the protein level.

Contrarily, significant *OsHKT2;4* expression was observed in the SNG leaf sheath and leaf blade under saline-alkaline conditions (Table 2). OsHKT2;4 is a co-K^+^/Na^+^ transporter that displays high permeability to K^+^ compared to Na^+^ and is expressed in several cell types, including root hair and vascular parenchyma cells (Horie et al. 2008; Lan et al. 2010; Sassi et al. 2012). This may play an important role in K^+^ transport in SNG under saline-alkaline conditions.

Although many macronutrients and micronutrients accumulated less in both rice varieties under saline-alkaline conditions than in the control and saline conditions, their accumulation, such as Ca, Mg, P, Mn, Zn, and Cu under saline-alkaline conditions tended to be higher in SNG than in KSH (Figs. 4, 5). In addition to K, the greater accumulation of these cation and divalent metals in SNG probably also contribute to superior saline-alkaline tolerance in SNG. Indeed, transcriptome analysis revealed significant expression of *OsZIP* family members (*OsZIP4, OsZIP5, OsZIP8,* and *OsZIP9*), *OsNRAMP* family members (*OsNRAMP5* and *OsNRAMP6*), and *OsIRTs* (*OsIRT1* and *OsIRT2*), which are involved in Zn, Mn, and/or Fe transport, in SNG roots under saline-alkaline conditions (Table S3) (Huang et al. 2020; Ishimaru et al. 2005, 2006, 2012; Lee and An 2009; Lee et al. 2010a, b; Peris-Peris et al. 2017; Yang et al. 2020; Yeo et al. 1991). GO analysis showed significant enrichment of GO terms related to “transition metal ion transport,” “metal ion transport,” and “zinc ion transport” in SNG roots (Fig. S3b). In contrast, the Fe content in the leaf sheath and leaf blade of SNG under saline-alkaline conditions was lower than that in KSH (Fig. 5k, l), and only the genes related to strategy I Fe^2+^ acquisition systems (OsIRT1 and OsIRT2) were upregulated (Table S3). However, strategy I Fe^2+^ acquisition in rice is not complete because of its low Fe(III) reduction activity (Ishimaru et al. 2006; Masuda et al. 2019). According to our results and the previous results, SNG may not have a highly efficient Fe acquisition system. Li et al. 2016 reported that the high saline-alkaline tolerance of the tolerant variety Dongdao-4 resulted from highly efficient Fe acquisition mechanisms; however, in the case of SNG, it appears that these mechanisms do not exist despite its high saline-alkaline tolerance.

GO analysis revealed numerous saline-alkaline inducible genes in SNG categorized in the GO terms related to biological processes, including “diterpene phytoalexin metabolic process,” “phytoalexin metabolic process,” “protein amino acid phosphorylation,” “defense response,” “isoprenoid metabolic process,” “chitin catabolic process” and “tryptophan metabolic process” (Figs. S3, S4), implying complex stress responses and tolerance mechanisms to saline-alkaline stress in SNG. Among these, *Os04g0179200* and *Os04g0179100*, which encode OsSDR110C-MS1 and OsSDR110C-MS2, respectively, are involved in the biosynthesis of momilactone A, a diterpene phytoalexin (Kitaoka et al. 2016; Shimura et al. 2007). Momilactone content was strongly correlated with salt and drought tolerance in rice (Xuan et al. 2016). The upregulation of these genes may participate in saline-alkaline tolerance mechanisms in rice. In addition, tryptophan is a biosynthetic precursor of melatonin and is involved in the tolerance mechanisms of biotic or abiotic stresses, such as salt stress (Li et al. 2019). One of the upregulated DEGs, *Os03g0264400*, encodes anthranilate synthase alpha subunit 2 (OsASA2), an essential enzyme for tryptophan synthesis (Tozawa et al. 2001). In a previous study, exogenous tryptophan alleviated salt stress in *Solanum tuberosum* (Gull et al. 2023)*.* Similar to our results, some studies have reported that tryptophan metabolic processes are activated in plants under saline-alkaline stress (Li et al. 2020; Yue et al. 2021), indicating that tryptophan is possibly related to saline-alkaline tolerance mechanisms in rice.

In conclusion, this study revealed that high K^+^ acquisition mechanisms were displayed under saline-alkaline stress conditions in the novel saline-alkaline tolerant rice variety SNG. It is also implied that OsHAK17, which is the saline-alkaline inducible gene, may partially support the K^+^ acquisition system under saline-alkaline stress conditions. Moreover, SNG may be superior to some tolerance mechanisms, such as divalent metal transport mechanisms. Our findings provide new knowledge about saline-alkaline tolerance mechanisms and contribute to developing new tolerant rice cultivars.

### Supplementary Information

Below is the link to the electronic supplementary material.

Supplementary file1 (PDF 847 KB)

## Data Availability

The data that support the findings of this study are available from the corresponding author upon reasonable request.
