# Supplementary material for: Potassium transporter OsHAK17 may contribute to saline-alkaline tolerant mechanisms in rice (Oryza sativa)
Source: J Plant Res. 2024 Mar 1;137(3):505–20. doi: 10.1007/s10265-024-01529-0 (PMC11082038; doi:10.1007/s10265-024-01529-0)
Supplement: Supplementary file 1 — Supplementary file1 (PDF 847 KB) [file 10265_2024_1529_MOESM1_ESM.pdf]

**Title: The potassium transporter OsHAK17 may partially contribute to saline-alkaline tolerant mechanisms in rice (*Oryza sativa* L.)**

**Journal name**: Journal of Plant Research

**Authors:**

Mami Nampei[1], Hiromu Ogi[1], Tanee Sreewongchai[2], Sho Nishida[3,4], and Akihiro Ueda[1] (akiueda@hiroshima-u.ac.jp)

[1] Graduate School of Integrated Sciences for Life, Hiroshima University, Higashi-Hiroshima, Hiroshima, Japan

[2] Department of Agronomy, Faculty of Agriculture, Kasetsart University, Bangkok, Thailand

[3] Faculty of Agriculture, Saga University, Saga, Saga, Japan

[4] United Graduate School of Agricultural Sciences, Kagoshima University, Kagoshima, Kagoshima, Japan

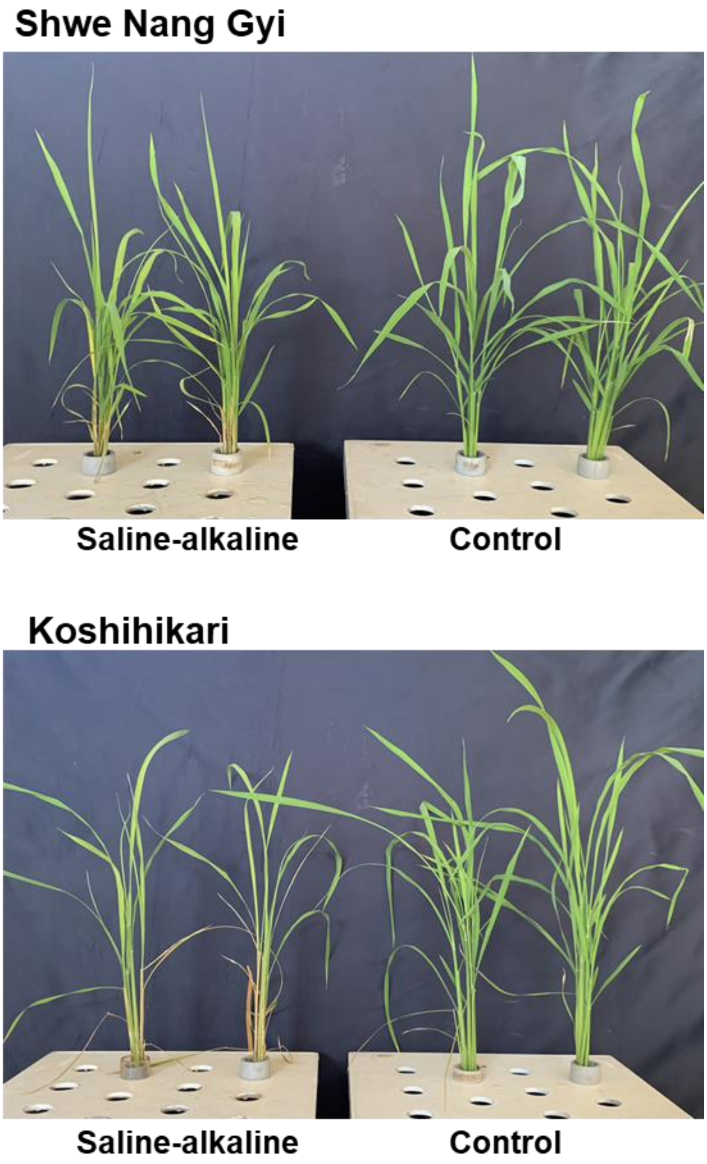


**Fig. S1** Growth of Saline-alkaline tolerant rice variety, Shwe Nang Gyi, and sensitive rice variety Koshihikari, under control and saline-alkaline (50 mM Na + pH 8.3) conditions. Six-week-old seedlings were subjected to saline-alkaline stress for 2 weeks.

**Table S1** List of primers for cloning.

| No. | Primer | Sequence |
|---|---|---|
| 1 | pDR195-F | CGGATCCAGCTTTGGACTT |
| 2 | pDR195-R | GCTCGAGGCTGGGGTATATT |
| 3 | OsHAK17-F | ACCCCAGCCTCGAGCCTTGTTGTTCCTCGCTCCTC |
| 4 | OsHAK17-R | CCAAAGCTGGATCCGCGATCAACATCGTAAGGCAAC |

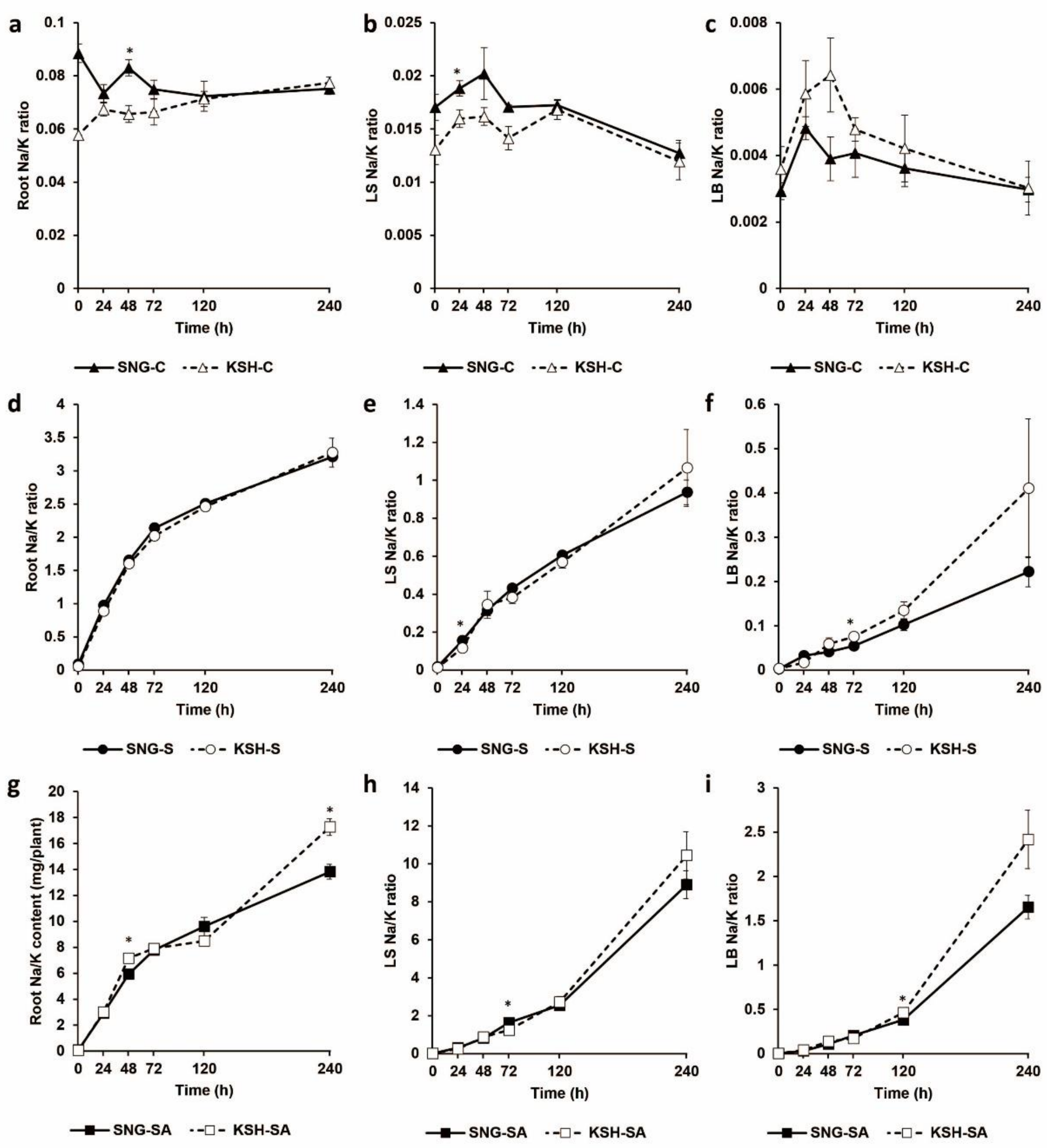


**Fig. S2** Na/K ratio under control (0 mM Na + pH 5.5) **(a-c)**, saline (50 mM Na + pH 5.5) **(d-f)**, and saline-alkaline (50 mM Na + pH 8.0-8.3) **(g-i)** conditions in the roots **(a, d, g)**, leaf sheaths **(b, e, h)** and leaf blades **(c, f, i)** of saline-alkaline tolerant variety (SNG) and saline-alkaline sensitive variety (KSH). The data is represented as the means of four replicates ± SE. The sodium contents were compared between the two rice varieties at each sampling time ($p$<0.05).

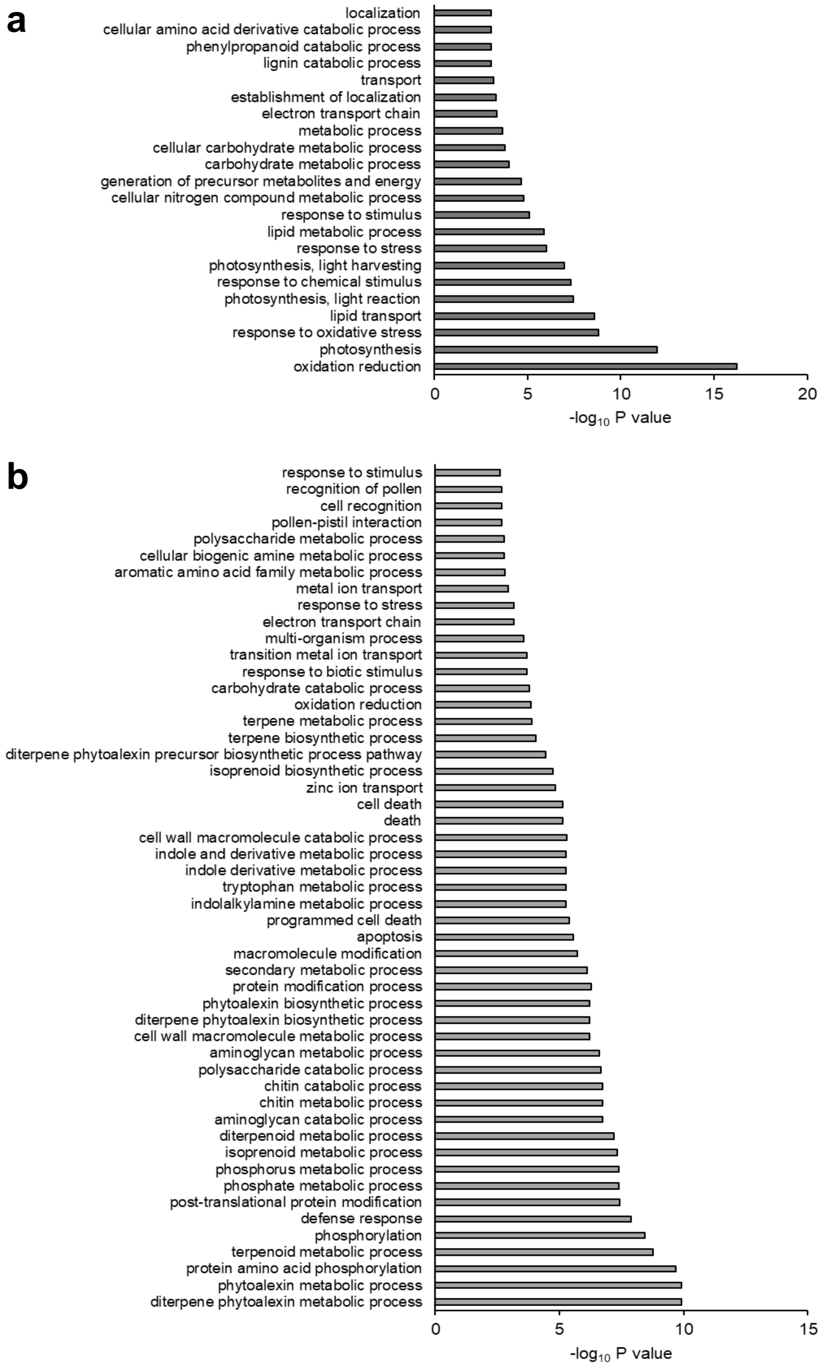


**Fig. S3** Significantly enriched GO terms by downregulated **(a)** and upregulated genes **(b)** in root during biological process in of the saline-alkaline tolerant variety, SNG. The False discovery rate (FDR) of enriched GO terms was < 0.05.

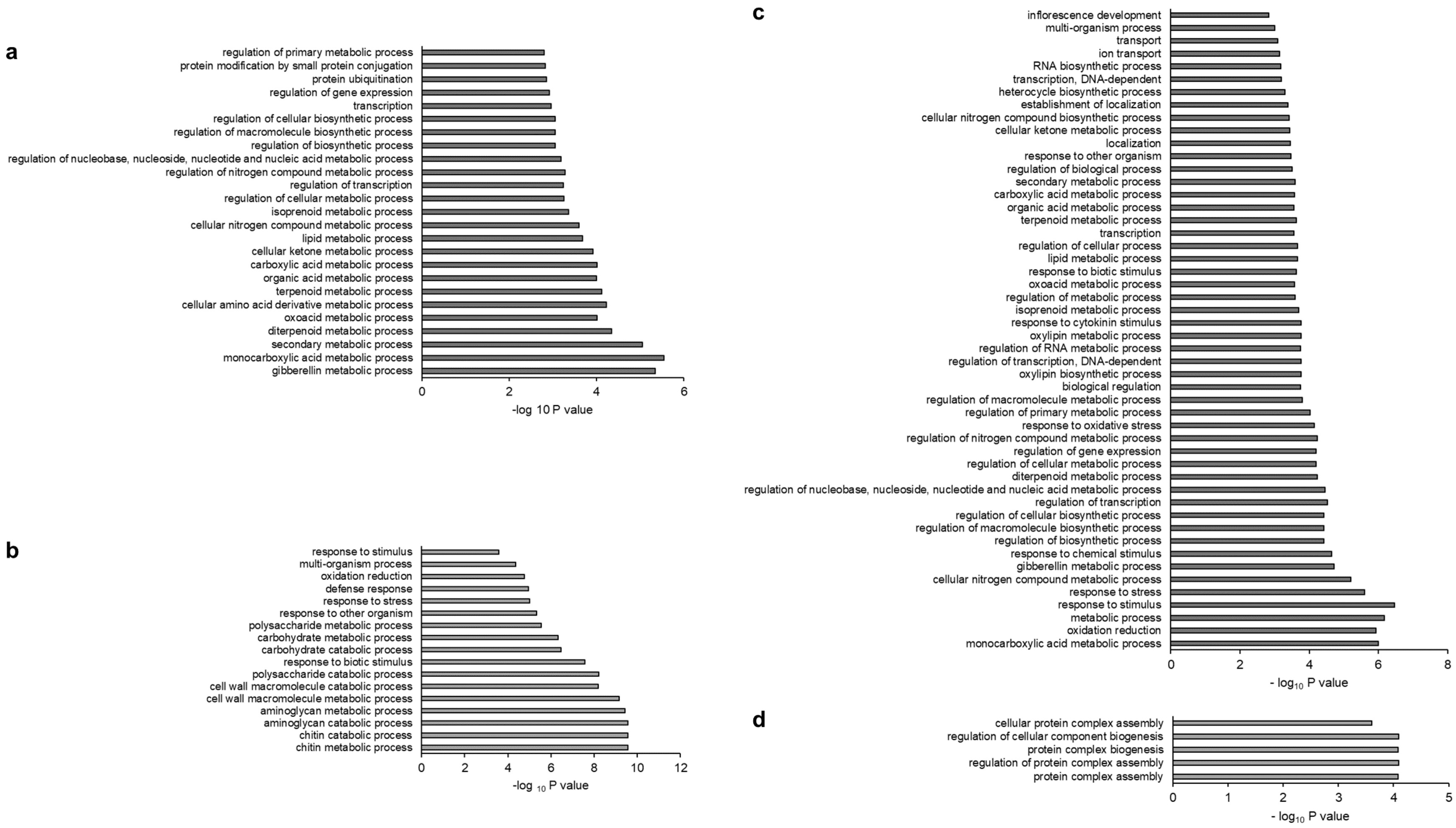
a
regulation of primary metabolic process
protein modification by small protein conjugation
protein ubiquitination
regulation of gene expression
transcription
regulation of cellular biosynthetic process
regulation of macromolecule biosynthetic process
regulation of biosynthetic process
regulation of nucleobase, nucleoside, nucleotide and nucleic acid metabolic process
regulation of nitrogen compound metabolic process
regulation of transcription
regulation of cellular metabolic process
isoprenoid metabolic process
cellular nitrogen compound metabolic process
lipid metabolic process
cellular ketone metabolic process
carboxylic acid metabolic process
organic acid metabolic process
terpenoid metabolic process
cellular amino acid derivative metabolic process
oxoacid metabolic process
diterpenoid metabolic process
secondary metabolic process
monocarboxylic acid metabolic process
gibberellin metabolic process
0
2
4
6
-log 10 P value
b
response to stimulus
multi-organism process
oxidation reduction
defense response
response to stress
response to other organism
polysaccharide metabolic process
carbohydrate metabolic process
carbohydrate catabolic process
response to biotic stimulus
polysaccharide catabolic process
cell wall macromolecule catabolic process
cell wall macromolecule metabolic process
aminoglycan metabolic process
aminoglycan catabolic process
chitin catabolic process
chitin metabolic process
0
2
4
6
8
10
12
-log 10 P value
c
inflorescence development
multi-organism process
transport
ion transport
RNA biosynthetic process
transcription, DNA-dependent
heterocycle biosynthetic process
establishment of localization
cellular nitrogen compound biosynthetic process
cellular ketone metabolic process
localization
response to other organism
regulation of biological process
secondary metabolic process
carboxylic acid metabolic process
organic acid metabolic process
terpenoid metabolic process
transcription
regulation of cellular process
lipid metabolic process
response to biotic stimulus
oxoacid metabolic process
regulation of metabolic process
isoprenoid metabolic process
response to cytokinin stimulus
oxylipin metabolic process
regulation of RNA metabolic process
regulation of transcription, DNA-dependent
oxylipin biosynthetic process
biological regulation
regulation of macromolecule metabolic process
regulation of primary metabolic process
response to oxidative stress
regulation of nitrogen compound metabolic process
regulation of gene expression
regulation of cellular metabolic process
diterpenoid metabolic process
regulation of nucleobase, nucleoside, nucleotide and nucleic acid metabolic process
regulation of transcription
regulation of cellular biosynthetic process
regulation of macromolecule biosynthetic process
regulation of biosynthetic process
response to chemical stimulus
gibberellin metabolic process
cellular nitrogen compound metabolic process
response to stress
response to stimulus
metabolic process
oxidation reduction
monocarboxylic acid metabolic process
0
2
4
6
8
- $\log_{10}$ P value
d
cellular protein complex assembly
regulation of cellular component biogenesis
protein complex biogenesis
regulation of protein complex assembly
protein complex assembly
0
1
2
3
4
5
- $\log_{10}$ P value

**Fig. S4** Significantly enriched GO terms by downregulated **(a)** and upregulated genes **(b)** in leaf sheaths and downregulated **(c)** and upregulated genes **(d)** in leaf blades during biological processes in the saline-alkaline tolerant variety, SNG. The false discovery rate (FDR) of enriched GO terms was < 0.05.

**Table S2** Relative expression level of sodium transporter genes in the root, leaf sheath, and leaf blade of saline-alkaline tolerant variety SNG under saline-alkaline conditions.

| | **Root** | | **Leaf sheath** | | **Leaf blade** | |
|---|---|---|---|---|---|---|
| Gene | $log_2FC$ | FDR | $log_2FC$ | FDR | $log_2FC$ | FDR |
| *OsSOS1* | -0.26 | 0.39 | 0.28 | 0.53 | 0.13 | 0.95 |
| *OsNHX1* | -2.29 | 0.00 | 0.52 | 0.47 | 0.90 | 0.03 |
| *OsNHX2* | 0.75 | 0.00 | 0.45 | 0.18 | 0.28 | 0.44 |
| *OsNHX3* | 0.09 | 1.00 | 0.06 | 1.00 | 0.37 | 0.24 |
| *OsNHX4* | -1.49 | 0.41 | -0.48 | 0.96 | -1.63 | 0.49 |
| *OsNHX5* | 0.08 | 1.00 | 0.22 | 0.75 | 0.16 | 0.79 |
| *OsHKT1;1* | -0.96 | 0.15 | -0.09 | 1.00 | 0.96 | 0.00 |
| *OsHKT1;3* | -1.14 | 0.23 | 0.19 | 1.00 | -1.03 | 0.01 |
| *OsHKT1;4* | -2.34 | 0.00 | -0.41 | 0.69 | 0.50 | 0.95 |
| *OsHKT1;5* | -0.18 | 0.93 | 0.96 | 0.08 | 1.18 | 0.10 |

**Table S3** Relative expression of divalent-metal transporters in the roots, leaf sheaths and leaf blades of saline-alkaline tolerant variety SNG under saline-alkaline conditions.

| | **Root** | | **Leaf sheath** | | **Leaf blade** | |
|---|---|---|---|---|---|---|
| **Gene** | $log_2FC$ | FDR | $log_2FC$ | FDR | $log_2FC$ | FDR |
| *OsNramp1* | 0.48 | 0.30 | -0.25 | 0.98 | -0.57 | 0.17 |
| *OsNramp2* | 0.82 | 0.00 | -0.23 | 0.80 | -0.23 | 0.63 |
| *OsNramp3* | 0.86 | 0.00 | -0.08 | 1.00 | -0.90 | 0.00 |
| *OsNramp4* | -0.93 | 0.00 | 0.80 | 1.00 | -0.58 | 0.77 |
| *OsNramp5* | 1.04 | 0.00 | -0.62 | 0.01 | -1.84 | 0.00 |
| *OsNramp6* | 1.15 | 0.00 | 0.08 | 1.00 | 0.13 | 1.00 |
| *OsNramp7* | 0.74 | 0.01 | 0.23 | 0.93 | 0.11 | 1.00 |
| *OsZIP1* | -0.75 | 0.00 | -1.63 | 0.00 | -6.96 | 0.02 |
| *OsZIP2* | 0.09 | 1.00 | 0.30 | 0.64 | -0.49 | 0.12 |
| *OsZIP3* | -0.45 | 1.00 | -0.07 | 1.00 | 0.00 | 1.00 |
| *OsZIP4* | 2.46 | 0.00 | 0.60 | 0.25 | -0.12 | 1.00 |
| *OsZIP5* | 2.44 | 0.00 | 0.00 | 1.00 | -0.64 | 1.00 |
| *OsZIP6* | 0.71 | 0.00 | -0.02 | 1.00 | -0.50 | 0.04 |
| *OsZIP7* | 0.82 | 0.03 | -0.72 | 0.32 | -1.03 | 0.09 |
| *OsZIP8* | 2.91 | 0.00 | 0.51 | 0.22 | -0.21 | 0.82 |
| *OsZIP9* | 2.11 | 0.00 | -1.17 | 1.00 | 0.00 | 1.00 |
| *OsZIP10* | 2.94 | 0.00 | -0.15 | 1.00 | -0.79 | 0.04 |
| *OsIRT1* | 2.77 | 0.00 | 0.10 | 1.00 | -0.94 | 0.26 |
| *OsIRT2* | 1.34 | 0.05 | 0.00 | 1.00 | 1.96 | 0.03 |
